# Measuring End-of-Life Care: Development of a National Questionnaire for Bereaved Family Members

**DOI:** 10.3390/healthcare14193178

**Published:** 2026-09-24

**Authors:** Alexandra Pereira, Amélia Ferreira, Sara Pinto

**Affiliations:** 1 School of Medicine and Biomedical Sciences of Abel Salazar Biomedical Institute, R. Jorge de Viterbo Ferreira 228, 4050-313 Porto, Portugal; maferreira@ulsts.min-saude.pt; 2 Community Care Unit of Lousada, Rua de Santo Tirso 70, 4620-848 Lousada, Portugal; 3 RISE-Health, Faculty of Medicine, University of Porto, Alameda Prof. Hernâni Monteiro, 4200-319 Porto, Portugal; sara.o.pinto@gmail.com

**Keywords:** bereavement, end-of-life care, family caregiver, health care questionnaires

## Abstract

Background: Evaluating end-of-life care quality remains a central challenge in health systems facing population ageing and complex chronic disease trajectories. Given the clinical challenges of direct data collection at the end-of-life, bereaved family members act as critical proxies, providing data on the care delivered in the final months of life. However, most available instruments lack the contextual sensitivity required for national-level epidemiological surveillance. Objectives: This study aims to develop and establish the face validity of a national questionnaire to evaluate the healthcare trajectory of adults with advanced chronic disease from the proxy perspective. Methods: A four-stage developmental protocol was implemented: (1) conceptual framework definition; (2) item generation through a review of national regulations, a scoping review of seven international instruments, and a focus group with clinical experts; (3) item reduction and refinement through expert panel review; and (4) face validity and feasibility testing through cognitive interviews and a pilot study. Results: The consensus process and pilot testing resulted in a final instrument comprising 84 items, structured into 7 sections and 17 thematic clusters. The questionnaire covers core care trajectories, the last 48 h of life, and bereavement support, while integrating specific national settings like integrated continuous care, alongside items on spiritual care and communication. The pilot study yielded a mean completion time of 14.6 min, while cognitive interview and pilot testing supported participant acceptability and feasibility for both telephone-assisted and self-administration modes. Conclusions: This questionnaire provides a face-validated, evidence-informed tool for post-death proxy reporting at the end-of-life. By aligning international quality indicators with country-specific healthcare structures, the instrument is ready for further testing of its reliability and validity and may support future comparative research, clinical auditing and health policy development.

## 1. Introduction

End-of-life care has become a cornerstone of modern healthcare systems. Despite the escalating demand, recent data from the OECD (2023) indicate that only half of those in need receive adequate support, often within fragmented systems that prioritize acute interventions over integrated palliative approaches [1]. This fragmentation frequently leads to burdensome transitions, overtreatment, and inefficient resource utilization, particularly through emergency department overreliance [2,3].

While integrating palliative care is proven to optimize outcomes and enhance cost-effectiveness [4,5,6], measuring end-of-life quality remains a complex challenge [7,8]. The inherent subjectivity of domains such as dignity and spirituality [7,9], coupled with the high prevalence of cognitive impairment among patients near death, demands the use of bereaved family members as proxies [10,11]. Retrospective proxy reports have thus emerged as a vital methodological tool to capture the lived experience of care [12].

However, a significant gap persists: most current metrics are conceptually heterogeneous and lack the sensitivity to account for diverse healthcare delivery models [13,14]. In many countries, quality indicators remain limited to administrative data, such as place of death or 30-day hospital readmissions, which fail to capture the nuances of care quality or patient and family expectations [1].

This evidence gap is particularly acute in countries facing advanced demographic transitions alongside highly centralized health systems. Portugal represents a compelling archetype of this challenge: it features one of the world’s most rapidly aging populations [15] and a disproportionate burden of chronic disease [16]. While recent initiatives have focused on refining the documentation and granularity of mortality data, such as the specific place of death [17], the actual clinical and social trajectories of these patients prior to death represent a critical knowledge gap [18]. Furthermore, standard international evaluation tools fail to map onto the country’s specific institutional framework, such as its unique integrated long-term and palliative care network [19], rendering foreign metrics structurally insensitive. Consequently, there is an urgent need for a culturally adapted, epidemiologically-oriented instrument.

This paper aims to describe the development and face validation of a national questionnaire designed to assess the healthcare received during the last months of life by adults with advanced chronic disease, from the perspective of the bereaved family member.

## 2. Materials and Methods

### 2.1. Study Design and Framework

The development of this national instrument followed a four-stage protocol adapted from Burns et al. (2008) [20]. This framework guided the processes of design, development, testing, administration, and dissemination (Table 1). To ensure high-quality reporting, we integrated the consolidated criteria for reporting qualitative research (COREQ) guidelines for the qualitative phase [21] and aligned the study with the COSMIN recommendations [22] for content validity.

### 2.2. Item Generation: Literature Review and Focus Group

We used a multi-method approach to generate items and ensure comprehensive coverage of the care context. First, we conducted a three-pronged review consisting of (1) Portuguese legal and technical documentation, (2) a systematic review of end-of-life care delivery in Portugal [18], and (3) a scoping review of international proxy-reported instruments.

Next, we conducted a focus group to capture clinical and practical nuances following COREQ guidelines [21]. The session was led by a female registered nurse from the research team (MSc, with extensive community-based palliative care experience). Although several participants knew the facilitator professionally, all were briefed on the researcher’s background and the study objectives. We used purposive sampling to recruit seven experts based on their professional backgrounds and end-of-life care expertise, including: nurses from primary, hospital and emergency care; a psychologist from long-term care; physicians from palliative and public health settings; and a nursing professor. 

The 90-min session was conducted online, audio-recorded, and observed by two additional researchers for field-note triangulation. Four open-ended questions explored care contexts, healthcare dimensions in the final three months and last 48 h of life, and other key end-of-life care attributes. Towards the end of the discussion, participants were asked whether they wished to add any further aspects that had not yet been addressed. No additional issues were raised. Because only one focus group was conducted, this procedure was used to assess whether further topics emerged within the group, rather than to claim thematic saturation across the wider target population. Following verbatim transcription and thematic content analysis by three members of the research team [23], areas, themes, and potential questionnaire items were identified. Formal inter-coder reliability or agreement statistics were not calculated; the researchers discussed the identified content and consolidated it into a common database. The resulting themes and items were subsequently returned to all focus group participants for member checking.

### 2.3. Item Reduction and Expert Panel Consensus

Before item reduction, all candidate items were labelled according to their source of origin. The research team subsequently mapped conceptually equivalent or closely related items across sources to characterize the degree of overlap in the initial item pool. This source-level mapping was used descriptively to examine the extent to which the different sources contributed overlapping or unique content. The resulting source-by-source overlap was summarized using frequencies and percentages.

The preliminary item pool was refined by a multidisciplinary expert panel (n = 6) to ensure the instrument’s clinical relevance and institutional alignment. The panel included three members of the research team and three representatives from the Portuguese Directorate-General of Health (one public health physician, one nurse, and one senior health technician).

Item reduction was conducted in three phases. First, potentially redundant or conceptually overlapping items were identified and discussed collectively by the panel using a structured binary assessment. Items were considered redundant when they addressed the same underlying aspect of care, within the same time period and care context, and were not expected to provide substantially different information to respondents. Items addressing related but distinct constructs were retained. 

For items considered potentially overlapping, the panel discussed whether they represented the same construct or distinct, complementary aspects of care. Where items were judged to capture essentially the same information, the panel retained the item considered most relevant to the intended domain, taking into account conceptual relevance, clarity, specificity, and respondent burden. Thus, exclusion was based on conceptual redundancy rather than similarity of wording alone.

Second, retained items were mapped into thematic sections according to the sections identified during the preceding qualitative and content-analysis stages. A minimum agreement threshold of 75% was required for final item retention. This process was designed to balance comprehensive content coverage with conceptual distinctiveness and respondent burden.

### 2.4. Questionnaire Formatting and Composition

In collaboration with a biostatistician, we refined the questionnaire to ensure technical integrity and visual clarity. Each item was reviewed to ensure person-first and socially sensitive language, avoiding technical jargon and complex terminology with the intent to accommodate different literacy levels. Abbreviations were removed to prevent ambiguity. Response scales were standardized using nominal, ordinal, or interval formats to facilitate both participant comprehension and subsequent statistical analysis. 

The research team finalized the composition to optimize response rates and ensure a logical flow. Two cover letters were developed, one for telephone administration and another for self-administration, each including the study objectives and an estimated completion time to manage respondent burden.

### 2.5. Pre-Testing and Pilot Study (Face Validity)

We conducted a two-stage validation process to assess feasibility and face validity. First, cognitive interviews were carried out with 10 individuals representing the target population to evaluate item interpretation, cognitive flow, and emotional appropriateness. Second, a pilot study was conducted with 30 bereaved family members, following international recommendations [24], who met predefined eligibility criteria regarding the time elapsed since the relative’s death. This phase provided evidence of the questionnaire’s face validity, ease of use of the response set, and estimated completion time, supporting its feasibility for future large-scale evaluation while indicating the need for further psychometric testing before national implementation.

### 2.6. Ethical Considerations

This study was approved by an Ethics Committee and conducted in accordance with the Declaration of Helsinki [25]. All participants provided informed consent. To ensure confidentiality and data protection, all responses were pseudonymized, and accessible exclusively to the research team. Given the sensitive nature of bereavement, the instrument’s formatting and administration protocols were specifically tailored to safeguard emotional well-being and minimize potential participant distress.

## 3. Results

The development process resulted in a comprehensive national instrument designed to evaluate the healthcare trajectory and quality of care during the last three months of life. An overview of the item generation and refinement phases is summarized in Table 2.

Figure 1 illustrates the item-tracking flow throughout the questionnaire development process, detailing the number of items generated from each evidence source, those removed at each stage and the items retained for subsequent refinement and validation.

### 3.1. Framework and Scope of the Instrument

The development process resulted in a multidimensional instrument designed for national surveillance of end-of-life care. The final specification defines the questionnaire’s scope across four consolidated domains: the clinical trajectory during the final three months of life, proxy perceptions of care quality, healthcare decision-making processes, and the adequacy of bereavement support. The instrument is operationalized for a target population of bereaved family members, utilizing an inclusive eligibility criterion that encompasses biological, legal, and meaningful non-kin relationships (e.g., close friends) with firsthand knowledge of the care provided to adult decedents (≥18 years) [26,27,28,29,30].

Regarding its application parameters, the instrument is standardized for a retrospective administration window between 3 and 12 months post-death. This temporal configuration ensures a balance between accuracy of clinical recall and the emotional stability required for participation in end-of-life research [27,31,32,33,34,35]. Although optimized for telephone-assisted interviews (including a formalized script for informed consent), the questionnaire’s structural architecture supports multimodal administration (online or postal), providing a versatile tool for long-term public health monitoring and clinical auditing.

### 3.2. Item Generation (Evidence Synthesis and Qualitative Inputs)

The item generation phase integrated findings from a comprehensive evidence synthesis and qualitative expert input, supporting the questionnaire’s contextual relevance and providing preliminary evidence of face validity.

The review of Portuguese legal and technical documentation identified four legal documents, two national strategic documents and one technical guideline (Table 3).

Although these documents establish the organizational basis of the National Palliative Care Network, they lack specific quality indicators or evaluation metrics for end-of-life care delivery. However, the analysis of the Death Certificate Information System (SICO) [42] enabled the derivation of three core variables: place of death, cause of death, and primary place of care. A major gap identified in this review was the absence of kinship or proxy contact information within the SICO system [42], which limits the identification of bereaved family members for research purposes.

The systematic review of healthcare delivery in Portugal [18] synthesized nine studies [43,44,45,46,47,48,49,50,51], mostly restricted to hospital settings. This review revealed significant gaps in the documentation of social, cultural and spiritual needs, as well as a limited coverage of non-pharmacological interventions, and after-death care. These findings informed seven themes, including non-hospital settings of care, social and cultural needs, psychological and spiritual needs, non-pharmacological interventions, after-death care, family care and emergency care episodes.

The scoping review (Appendix A) identified seven validated instruments for proxy assessment of end-of-life care: (1) the After-Death Bereaved Family Member Interview [27,52]; (2) the Bereaved Family Survey [35]; (3) Care Of the Dying Evaluation (CODE) [34]; (4) the Client-Centered Care Questionnaire [53]; (5) the Family Assessment of Treatment at End-of-life (FATE) [32]; (6) the Toolkit of Instruments to Measure End-of-Life Care (TIME) [31]; and (7) the Views of Informal Carers—Evaluation of Services Short Form (VOICES-SF) [33]. The synthesis of these instruments focused on several critical parameters, including country of origin, structural dimensions, number of items, recruitment methodologies, and the specific timing of post-death administration (detailed in Appendix B). The comparative analysis of these instruments yielded a preliminary pool of 216 items grouped into 37 thematic clusters. 

### 3.3. Focus Group Findings

The focus group provided critical clinical depth, contributing to the translation of theoretical domains into 13 themes and 58 specific items. The seven participants represented diverse professional backgrounds and care settings and highlighted the need to include home care, hospital units, nursing homes, and the National Network of Integrated Continuous Care (RNCCI). Within home-based care, participants emphasized the importance of distinguishing between family health teams, integrated continuous care teams, and community palliative support teams.

The discussion also helped refine the distinction between the “last three months” and the “last 48 h” of life. For the final 48 h, participants highlighted symptom management, dignity, emergency care, and the circumstances surrounding the time and place of death. Advance directives, communication regarding the time of death, and bereavement support were also identified as relevant domains.

During the discussion, participants were asked whether they wished to add further aspects; no additional issues were raised. Given that saturation was assessed within a single focus group, this finding should be interpreted as an indication that no further domains emerged in this group rather than as evidence of thematic saturation across the broader population. The responses were transcribed and reviewed by three members of the research team, who identified the themes and corresponding items through content analysis. Formal inter-coder agreement was not calculated.

In the following week, participants received the transcripts, field notes, and database containing the proposed themes and questions for member checking. All seven participants confirmed the material without requesting corrections or additions. No participants declined the invitation or withdrew from the study.

### 3.4. Item Reduction

A total of 277 candidate items were generated from the different evidence sources before item reduction. To characterize the contribution of each source, the provenance of each candidate item was mapped and conceptual overlap between sources was examined prior to the panel-based item reduction process. Of the 58 items generated through the focus group, 32 (55.2%) represented unique content that was not identified in the other evidence sources, while 26 (44.8%) overlapped conceptually with content identified through the scoping review. No conceptual overlap was identified between items derived from the legal and regulatory documents and those generated from the other evidence sources.

These findings indicate that the different evidence sources contributed complementary information to the initial item pool. The scoping review provided the main evidence base for the initial set of items, while the focus group contributed substantial additional content, with more than half of its items representing domains or aspects not identified in the other sources. The legal and regulatory documents further contributed context-specific content that was not represented in the scoping review or focus group.

Following this source-level mapping, the panel assessed the 277 candidate items for conceptual redundancy and overlap. A total of 177 items were excluded primarily because they were judged to be redundant or conceptually overlapping, while the remaining 100 items were retained for subsequent refinement and validation.

After a further 18 items that were not applicable to the organizational context of the Portuguese healthcare system were excluded, the pool was reduced from 100 to 82 items across 17 themes. During this consensus phase, seven items had their response options modified or added (Questions 1, 9, 49, 55, 59, 60, and 69), and the final structure was organized into seven sections: (1) characterization; (2) healthcare received in the last three months of life; (3) healthcare received in the last two days of life; (4) circumstances at the time of death; (5) advance directives; (6) bereavement support; and (7) overall quality and satisfaction with healthcare. 

### 3.5. Formatting and Composition

Technical refinement led to the replacement of technical jargon with lay terminology and the minimization of abbreviations. Response options were standardized across the questionnaire using nominal, ordinal, or interval formats to improve clarity and support subsequent statistical analysis. 

In addition, the structure of the questionnaire was adapted to account for the different organizational origins of palliative care units, including central hospitals, private hospitals, and units within the RNCCI, in order to facilitate the analysis of the patient’s clinical trajectory and access to palliative care. The panel also decided to split Question 67 to enhance understanding of the phenomenon under study.

The visual layout was finalized with a logical routing system designed to reduce respondent burden and cognitive effort. Two versions of the cover letter were produced, one for telephone administration and one for self-administration. Based on initial simulations, the estimated completion time was approximately 13 min.

### 3.6. Pre-Testing and Face Validity

Cognitive interviews led to the refinement of 15 items (Questions 13, 16, 18, 27, 33, 41, 45, 46, 51, 54, 56, 58, 61, 64 and 68), mainly through the addition of specific response options. In addition, four participants suggested the inclusion of an open-ended question to provide additional comments or suggestions related to the recent experience of the bereaved family; this became the 84th and final question of the questionnaire. The pilot study confirmed the questionnaire’s face validity and feasibility for both telephone and self-administration. The mean completion time in the pilot phase was 14.6 min, and no further modifications were required. The final National Questionnaire on End-of-Life Care comprises 84 items organized into seven sections and 17 themes (Figure 2). The first section (A. Characterization) was primarily used to organize the information and did not contain any questions of its own.

## 4. Discussion

The systematic evaluation of end-of-life care has gained increasing importance globally, driven by the need to shift from purely volume-based health metrics to patient and family-centered quality indicators [5,6,7,8,9,10,11,12,13,14,15,16,17,18,19,20,21,22,23,24,25,26,27,28,29,30,31]. The development and face validation of this national questionnaire represent an important methodological step toward the future evaluation of end-of-life care within the Portuguese healthcare system. By capturing the comprehensive healthcare trajectory through proxy reporting, this instrument addresses a long-standing reliance on crude administrative metrics (such as hospital mortality rates) which may fail to reflect the actual quality of care or the lived experiences of patients and their families [5,6,7,8,9,10,11,12,13,14,15,16,17,18,19,20,21,22,23,24,25,26,27,28,29,30,31,32,33,34,35,36,37,38,39,40,41,42,43,44,45,46,47,48,49,50,51,52,53,54].

By adopting the Burns et al. (2008) framework [20], this study ensured a rigorous transition from conceptual item generation to feasibility testing, resulting in a tool that aligns with international quality standards [22] while maintaining high contextual sensitivity. A major strength of this multi-stage process was the integration of diverse evidence sources. This holistic and global synthesis directly addresses a historical deficit in Portuguese end-of-life research, which has traditionally focused on physical symptom management while neglecting social, spiritual, and non-pharmacological interventions [18]. By embedding these dimensions into a nationally developed framework, the questionnaire aligns with recent international mandates, such as those from the Lancet Commission on the Value of Death [55], which calls for a rebalancing of healthcare systems toward compassionate, multidimensional end-of-life care. The convergence between international quality indicators derived from well-established tools such as the VOICES-Short Form [33] and TIME [31], and the qualitative nuances provided by local and national experts contributed to content development and face validity. Importantly, international instruments often lose clinical utility when directly translated without institutional adaptation. Considering this, the explicit inclusion of specific care settings, such as the RNCCI and diverse community-based palliative teams, reflects the unique organizational architecture of the Portuguese National Health Service. This structural alignment supports the instrument’s ability to map complex patient transitions across acute, continuous, and community care sectors, avoiding the lack of sensitivity common to unadapted foreign tools.

From a clinical perspective, the final structure of 84 items across seven sections captures the end-of-life trajectory from the last three months to the bereavement period. The chronological distinction between the “last three months” and the “last 48 h” of life is especially relevant. It allows researchers and clinicians to isolate phase-specific systemic failures, distinguishing between chronic care coordination deficits in the preceding months and acute communication or symptom-control failures during the active dying phase. The pilot study provided evidence supporting the instrument’s feasibility and face validity. In bereavement research, minimizing respondent burden is paramount to mitigating study attrition and ensuring ethical safety [8,56]. A mean completion time of 14.6 min, combined with the flexible multimodal design (telephone and self-administration), suggests a relatively low respondent burden and supports the questionnaire’s potential for use in future large-scale studies and quality assessment initiatives, subject to further psychometric evaluation. Furthermore, the established retrospective administration window of 3 to 12 months post-death strikes a critical methodological balance documented in international literature: it allows sufficient time for the acute grieving process to stabilize, thereby reducing participant distress, while preserving the cognitive recall accuracy required for robust data collection [57].

Finally, the close involvement of the Directorate-General of Health throughout the co-design and validation phases strengthens the instrument’s contextual relevance and its potential for future systemic integration. This collaboration bridges the gap between methodological development and public health execution [58], providing a foundation for further testing and validation of the questionnaire and for evaluating its potential future contribution to clinical auditing, cross-regional comparisons, and evidence-informed health policy aimed at safeguarding dignity at the end of life. Beyond its national application, the architecture of this validation process offers broader, transferable lessons for the international community engaged in end-of-life quality monitoring. The field-testing phase demonstrated that evidence supporting face validity and structural depth can coexist within a strict 15-min administrative window, effectively bypassing a common tension in bereavement research between data granularity and respondent burden. This operational efficiency is clinically significant, as it addresses the frequent ethical concern of clinician ‘gatekeeping’ [59], where healthcare providers withhold proxy access out of fear of exacerbating family distress. Furthermore, the identification of the administrative gap within the electronic death registry (SICO) [60], which registers clinical data but omits proxy contacts, serves as a cautionary lesson for international health systems. It underscores that the global push toward sophisticated digital death certification must be matched by cross-institutional protocols that ethically bridge epidemiological mortality tracking with active quality-of-care auditing.

### Strengths, Limitations and Future Perspectives

A key strength of this study lies in the multi-perspective, co-design approach utilized throughout the development process. By intentionally involving a diverse group of stakeholders, including biostatisticians, public health authorities, multiprofessional clinical experts, and bereaved family members, we ensured evidence contribution to content validity and cross-disciplinary alignment from the outset. Furthermore, the strict alignment with international reporting guidelines provides a high degree of methodological transparency, establishing a reproducible blueprint for instrument adaptation in vulnerable populations.

However, some limitations must be acknowledged. First, the absence of direct participation from established patient and caregiver associations may have limited the co-creation process and the integration of broader user-centered advocacy perspectives. In addition, while the inclusive and legally broad definition of “family” adopted in this study maximizes epidemiological reach, it may introduce variability in proxy reporting accuracy, although this remains a recognized and accepted trade-off in end-of-life research [27]. 

Only one focus group was conducted. However, the purposive inclusion of participants with diverse professional backgrounds and experience across different care settings may have enhanced the breadth of perspectives captured; nevertheless, additional focus groups might have identified further or alternative views. The findings from the focus group should therefore be understood primarily as an exploratory and content-generating contribution to questionnaire development rather than as an exhaustive account of all relevant domains of end-of-life care.

A further limitation related with the focus group concerns the relationship between the facilitator and the participants. The facilitator was a registered nurse with previous experience in palliative care, and most participants knew her professionally before the focus group. This clinical experience may have facilitated the discussion by providing contextual understanding and enabling the facilitator to explore issues relevant to end-of-life care. However, pre-existing professional familiarity may also have influenced participants’ contributions, for example through social desirability, greater agreement with the facilitator’s perspectives, or reluctance to express divergent views. In addition, no formal inter-coder agreement assessment was performed during the qualitative content analysis; coding decisions were instead discussed and consolidated by the research team.

The 75% threshold applied during item reduction reflected agreement regarding item retention should not be interpreted as a quantitative measure of content validity. Consequently, a formal item-level Content Validity Index could not be calculated from the available data. Therefore, the absence of new topics within this session should not be interpreted as evidence of thematic saturation across the wider population of professionals involved in end-of-life care.

Finally, as this study describes the initial development and face validation phases, full psychometric testing, including internal consistency, structural validity, and construct validation, has yet to be performed.

Despite these limitations, the questionnaire provides a rigorous and structurally stable foundation for subsequent large-scale psychometric evaluation. Its applicability is highly transferable and may extend beyond the national context to other countries with similar universal access, tax-funded national health services, particularly within Southern Europe, which share comparable demographic and institutional architectures. Future research should focus on the widespread national implementation of the instrument to establish the first population-based quality indicators, monitor patient clinical trajectories, and identify regional disparities in end-of-life care delivery to directly inform public health resource allocation.

## 5. Conclusions

This study provides a methodological pathway for the development and face validation of the first national instrument dedicated to assessing the quality of end-of-life care. By integrating international quality standards with an analysis of national legal and clinical frameworks, the resulting questionnaire captures the multifaceted nature of the healthcare trajectory from the unique perspective of bereaved family members.

The instrument has a comprehensive scope, encompassing clinical, social, and spiritual dimensions across different care settings. Its feasibility for telephone and self-administration and its relatively low respondent burden support its potential for use in future large-scale studies and quality assessment initiatives. However, further evaluation of its reliability, construct validity, and performance in larger and diverse samples is needed before its use in epidemiological surveillance, clinical auditing, or policy development can be recommended.

While contextually tailored to Portugal, the methodological approach may provide a useful framework for other countries seeking to develop culturally sensitive instruments for evaluating end-of-life care. Further testing and validation of the questionnaire will be essential to determine its applicability across settings and to establish its value for generating evidence to support quality improvement, clinical practice, and health policy. Ultimately, this work contributes to the broader effort to improve the visibility and quality of care provided during the final stages of life, with a focus on the dignity of patients and their families.

## Figures and Tables

**Figure 1:**
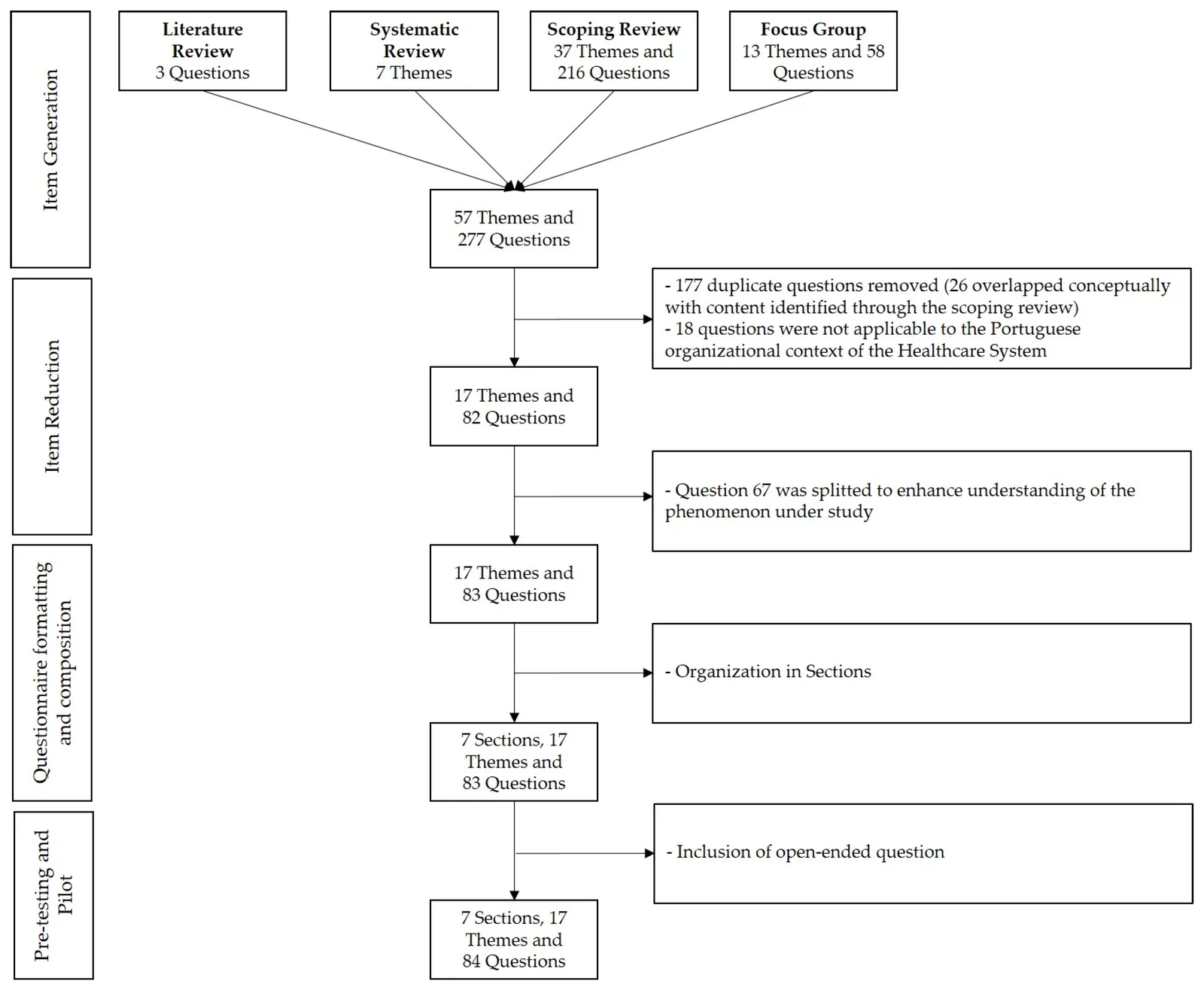
Item-tracking flowchart of the questionnaire development.

**Figure 2:**
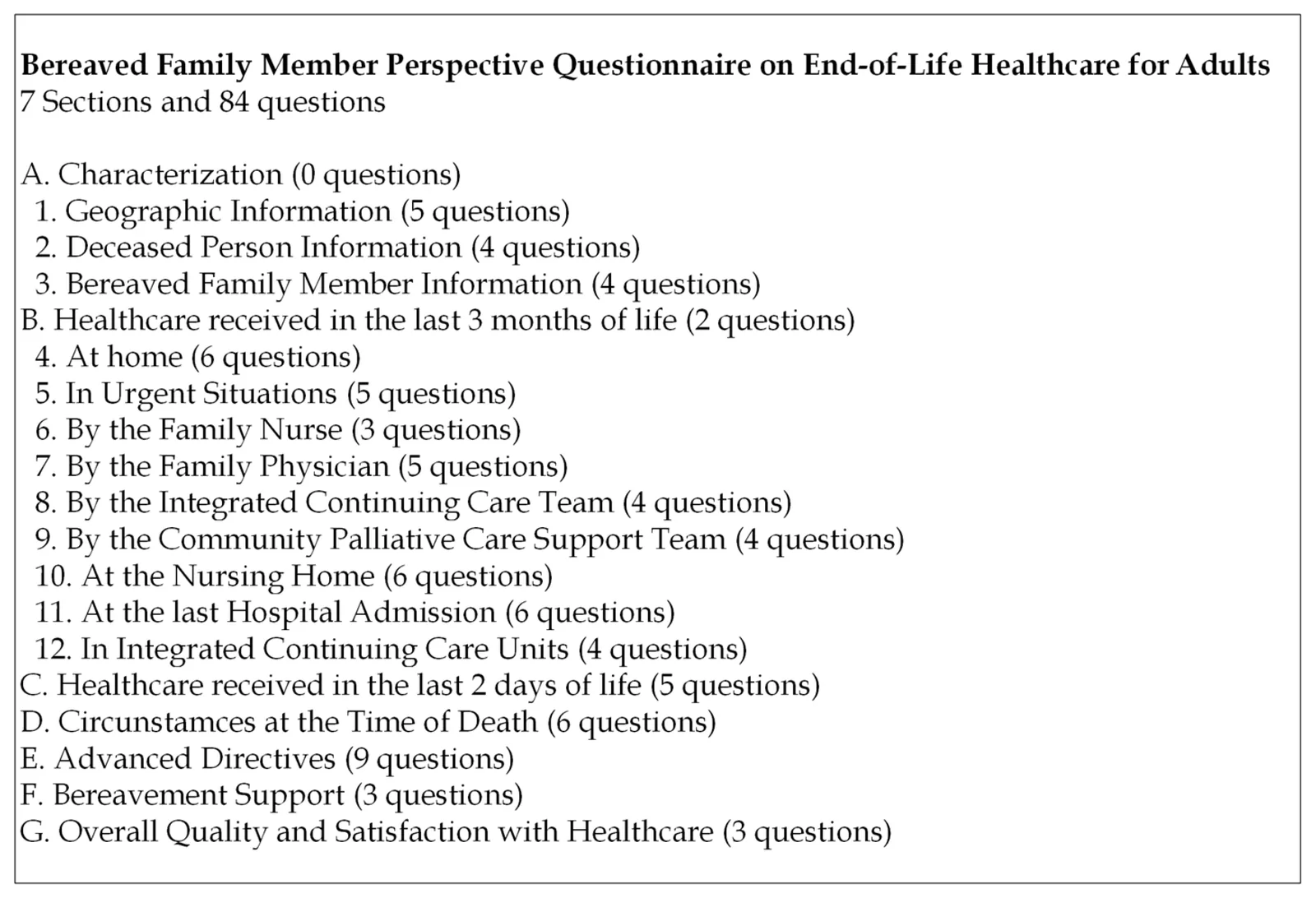
Final version of the Questionnaire.

**Table 1:** Overview of the questionnaire development and testing process.

Area	Step	Purpose
Design	Determining the objective	To specify research questions, the topic in analysis and respondents.
	Identifying the sampling frame	To identify the sampling method, that is the respondents from whom information is collected and analyzed.
Development	Item generation	To systematically consider all potential items for inclusion in the questionnaire, with the objective of addressing the key domains identified by the research questions.
	Item reduction	To reduce the number of potentially relevant items in order to minimize respondent burden.
	Questionnaire formatting	To ensure that questions are phrased in a socially and culturally sensitive manner, using clear and accessible language, and to develop succinct and unbiased response formats.
	Questionnaire composition	To create a positive initial impression, enhance visual appeal, and increase response rates.
	Pre-testing	To assess whether respondents interpret the questions consistently and to evaluate the appropriateness of each included item.
Testing	Pilot-testing	To evaluate the overall performance of the questionnaire, as well as its face and content validity.

Adapted from Burns et al. 2008 [20].

**Table 2:** Summary of the instrument development phases and item yield.

Area	Step	Method	Results
Development	Item generation	Literature review	Review of legal and technical documents: 3 questions Systematic review: 7 themes Scoping review: 7 instruments, 37 themes and 216 questions
		Focus Group	13 themes and 58 questions
	Item reduction	Expert Panel	Reduced 57 themes and 277 questions to 17 themes and 82 questions (68 from literature review and 14 from focus group). Of the 277 candidate items, 177 were removed because they were redundant or conceptually overlapping, leaving 100 items; a further 18 were excluded because they were not applicable to the organizational context of the Portuguese healthcare system, resulting in 82 retained items.
	Questionnaire formatting	Expert Panel and Biostatistician Analysis	Response options for seven questions response options were added or modified (Questions 1, 9, 49, 55, 59, 60, and 69). The panel also decided to split Question 67 to enhance understanding of the phenomenon under study. The themes (n = 17) and questions (n = 83) were organized in 7 sections.
	Questionnaire composition	Research Team Analysis	Two cover letters were created (telephone interview and self-administration). The questionnaire was formatted to improve readability and ease of completion.
	Pre-testing	The questionnaire was administered to 10 participants who shared characteristics with the target population.	Participants suggested improvements to 15 different questions (Questions 13, 16, 18, 27, 33, 41, 45, 46, 51, 54, 56, 58, 61, 64 and 68. An open-ended question was also added to allow participants to provide additional comments or suggestions related to their recent experience as bereaved family members. The final questionnaire comprised 7 sections, 17 themes and 84 questions. Participant characteristics: Age: 61.8 years (mean; SD: 8.0) Sex: 80.0% female; 20.0% male Educational level: 50.0% primary education; 30.0% secondary education; 20.0% higher education. Relationship with deceased: 70.0% partner; 30.0% son Care settings: 10.0% nursing home; 80.0% home and hospital; 10.0% home, hospital and nursing home Region: 40.0% North; 10.0% Centre; 30.0% Lisbon and Tagus Valley; 10.0% Alentejo; 10.0% Algarve Time after death: 303.2 days (mean; SD: 39.0) Dropouts: 0; Refusals: 0
Testing	Pilot	The questionnaire was administered to 30 bereaved family members.	Participant characteristics: Age: 60.7 years (mean; SD: 14.9) Sex: 80.0% Female; 20.0% male Educational level: 60.0% primary education; 16.7% secondary education; 23.3% higher education. Relationship with deceased: 40.0% partner; 46.7% son; 13.3% other relationship Care settings: 6.7% home; 3.3% home and nursing home; 10.0% nursing home; 6.7% hospital; 6.7% RNCCI; 50.0% home and hospital; 3.3% home, hospital and RNCCI; 3.3% home, hospital and nursing home Region: 36.7% North; 13.3% Centre; 23.3% Lisbon and Tagus Valley; 13.3% Alentejo; 13.3% Algarve Mode of administration: 36.7% self-administration; 63.3% telephone Completion time: 14.6 min (mean; SD: 3.4) Time after death: 298.3 days (mean; SD: 38.7) Dropouts: 0; Refusals: 0.

**Table 3:** Legal and technical documentation included in the conceptual framework.

Type of Document	Document
Legal Document	Decree-Law No. 101/2006 of 6 June, which establishes the National Network for Integrated Continuous Care [36]
	Law No. 25/2012 of 16 July, which establishes the Legal Framework for Advance Directives (Living Will) [37]
	Law No. 52/2012 of 5 September, which establishes the Basic Law on Palliative Care [38]
	Ordinance No. 340/2015 of 8 October, which regulates, within the scope of the National Palliative Care Network (RNCP), the characterization of services, admission to local teams, and the conditions and safety requirements for the construction of palliative care facilities [39]
National Health Strategic Document	National Palliative Care Program [40]
	Strategic Plan for the Development of Palliative Care 2017–2018 [41]
Technical Guideline	Guideline No. 020/2013 from the Directorate-General of Health: Electronic Death Certificate—Use of the Death Certificate Information System (SICO) [42]

**Figure A1:**
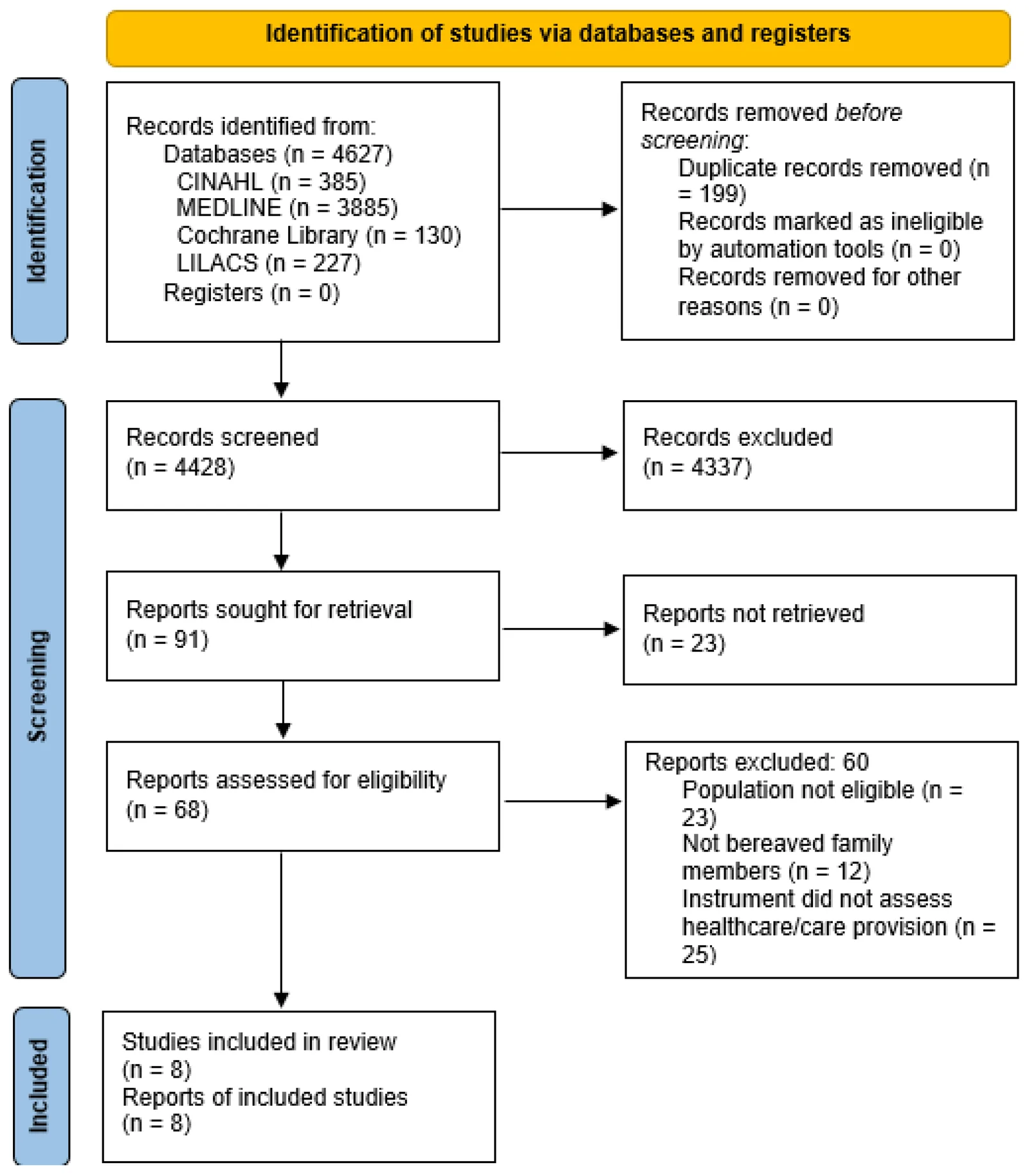
PRISMA-ScR flow diagram. (Adapted from: BMJ (2021) [61]).

## Data Availability

The questionnaire developed in this study is not publicly available. Access is restricted to help preserve the integrity and version control of the instrument, prevent the circulation or use of modified or outdated versions, and support its appropriate application in research and clinical contexts. The questionnaire is available from the corresponding author upon request.

## Abbreviations

The following abbreviations are used in this manuscript:

CODE	Care Of the Dying Evaluation
COREQ	Consolidated Criteria for Reporting Qualitative Research
FATE	Family Assessment of Treatment at End-of-life
RNCCI	National Network of Integrated Continuous Care
SICO	Portuguese Electronic Death Registry
VOICES-SF	Views of Informal Carers—Evaluation of Services Short Form

## Appendix A

### Appendix A.1. Scoping Review Design

The review was structured according to the Population–Concept–Context framework and conducted in accordance with established methodological guidance for scoping reviews. The reporting of the review followed the Preferred Reporting Items for Systematic Reviews and Meta-Analyses extension for Scoping Reviews (PRISMA-ScR).

### Appendix A.2. Scoping Review Question and Objectives

The review was guided by the following research question: “What instruments have been used to characterize healthcare provided to adults at the end of life, from the perspective of bereaved family members, regardless of the care setting?”. The review aimed to identify and map the instruments used to characterize the healthcare received by adults at the end-of-life from the perspective of bereaved family members, regardless of the care setting.

The PCC framework was defined as follows:

- Population: adults (aged ≥ 18 years) at the end of life or deceased adults whose end-of-life care was retrospectively assessed;
- Concept: instruments used to assess or characterize healthcare provided during the end-of-life period;
- Context: any healthcare setting or place of care;
- Perspective: bereaved family members acting as respondents or informants.

The specific objectives were:

- To identify the instruments used to assess or characterize healthcare provided to adults at the end of life from the perspective of bereaved family members.
- To describe the dimensions and domains of healthcare assessed by the identified instruments, including, where applicable, satisfaction with care, communication, decision-making, symptom management, family involvement, continuity and coordination of care, among others.
- To characterize the reference period of care assessed by each instrument, including the period of the patient’s end-of-life trajectory to which the assessment refers.
- To characterize the timing of instrument administration following the patient’s death, including the interval between death and data collection.
- To identify the methods used to administer the instruments, including face-to-face administration, telephone interviews, postal questionnaires, email, online platforms, or other methods of data collection.
- To characterize the populations and healthcare settings in which the instruments have been used, including the characteristics of bereaved family respondents and the contexts in which end-of-life care was provided.

### Appendix A.3. Eligibility Criteria

Studies were eligible for inclusion if they: (1) included adults (aged ≥ 18 years) who were at the end of life or had died following a period of chronic disease; (2) involved bereaved family members as respondents or informants; (3) used, developed, validated, adapted, or evaluated an instrument designed to assess or characterize healthcare provided to the deceased adult during the end-of-life period; (4) assessed one or more dimensions related to healthcare provision, such as clinical pathways, satisfaction with care, quality of care, communication, decision-making, involvement in care, symptom management, continuity or coordination of care, or support provided to patients and/or families; (5) were conducted in any healthcare setting, without restriction regarding the place of care and (6) published until 2016.

Studies were excluded if they: (1) focused exclusively on children or adolescents; (2) included assessments conducted only by patients, healthcare professionals, or non-bereaved caregivers; (3) assessed exclusively bereavement outcomes, grief, psychological distress, or quality of life of bereaved family members, without evaluating healthcare provided to the deceased; (4) used instruments assessing only symptoms, clinical status, or quality of life of the patient without assessing healthcare provision; (5) assessed only the quality of dying or death without a component related to healthcare provision; (6) merely mentioned an instrument without using or providing relevant information about its application; or (7) were editorials, letters to the editor, commentaries, study protocols, or conference abstracts lacking sufficient information for data extraction.

No restrictions were imposed on the reference period of care assessed by the instrument, the interval between the patient’s death and instrument administration, or the method of instrument administration, as these characteristics constituted variables of interest in this review. Studies reporting the translation, cultural adaptation, validation, or psychometric evaluation of an existing instrument were considered eligible when they met the remaining inclusion criteria. No language restriction was also used.

### Appendix A.4. Information Sources and Search Strategy

A comprehensive literature search was conducted across four electronic databases: CINAHL, MEDLINE, LILACS, and the Cochrane Library.

The search strategy was developed according to the PCC framework and combined controlled vocabulary, where applicable, with free-text terms related to end-of-life care, bereaved family members, healthcare assessment, and measurement instruments. Search terms and syntax were adapted to the specific requirements and controlled vocabularies of each database.

The search strategy was designed to retrieve studies addressing instruments used by bereaved family members to retrospectively assess healthcare provided to adults during the end-of-life period. Reference lists of included studies and other relevant publications were also examined to identify additional potentially eligible studies.

### Appendix A.5. Study Selection

All records retrieved from CINAHL, MEDLINE, LILACS, and the Cochrane Library were exported and combined into a single database. Duplicate records were identified and removed before the screening process.

Study selection was conducted in two sequential stages. First, titles and abstracts were screened against the predefined eligibility criteria. Records that clearly failed to meet the inclusion criteria were excluded. When eligibility could not be determined from the title and abstract alone, the record was retained for full-text assessment.

Second, the full texts of potentially eligible studies were retrieved and assessed against the inclusion and exclusion criteria. Studies were included when they reported the use, development, validation, adaptation, or evaluation of an eligible instrument completed by bereaved family members to assess or characterize healthcare provided to an adult during the end-of-life period.

Reasons for exclusion were recorded during full-text assessment and categorized according to the eligibility criteria. Potential reasons included an ineligible population, absence of bereaved family members as respondents, absence of an eligible instrument, assessment of outcomes unrelated to healthcare provision, absence of an end-of-life context, or insufficient information to establish eligibility.

Any uncertainties regarding study eligibility were resolved through discussion and consensus. The study selection process, including the number of records identified, duplicates removed, records screened, full-text articles assessed, studies excluded with reasons, and studies ultimately included, was documented using a PRISMA flow diagram (Figure A1).

### Appendix A.6. Data Extraction

A standardized database was developed, using Microsoft Excel, to systematically extract relevant information from the included studies.

For each included study, the following information was extracted, when available:

- author(s), year of publication, and country;
- study aim and methodological design;
- characteristics of the deceased population;
- characteristics and relationship of the bereaved family respondent to the deceased;
- healthcare setting and place of care;
- name and version of the instrument;
- purpose of the instrument;
- dimensions or domains of healthcare assessed by the instrument;
- reference period of care assessed by the instrument;
- interval between the patient’s death and administration of the instrument;
- method of instrument administration.

### Appendix A.7. Results

Table A1 and Table A2 (Appendix B) summarize the results of the scoping review.

**Table A1:** Studies Included in the Scoping Review (unpublished data).

Authors, Year of Publication	Study Objectives	Study Design	Identification of Participants	Participants	Instrument
Teno, et al., 2004 [31]	To evaluate the quality of end-of-life care in the United States across different care settings from the perspective of bereaved family members, and to compare family perceptions of care according to the patient’s last place of care.	National cross-sectional mortality follow-back survey.	Participants were identified using information recorded on the death certificate.	1578 participants	Toolkit of Instruments to Measure End-of-Life Care
Casarett, et al., 2008 [32]	To develop and evaluate the Family Assessment of Treatment at End-of-Life as a nationwide quality measure for palliative and end-of-life care in the U.S. Department of Veterans Affairs.	Multicenter observational validation study (cross-sectional bereavement survey).	Participants were identified through the Veterans Affairs’ electronic medical record system.	1026 participants	Family Assessment of Treatment at End of Life Survey
Brazil, et al., 2012 [53]	To evaluate the patient-centeredness of community palliative care from the perspective of family caregivers who had cared for a terminally ill family member receiving formal home care services.	Cross-sectional survey study.	Participants had been designated as receiving palliative care and had received formal home care services by a Community Care Access Centre.	111 participants	Client-Centered Care Questionnaire
Gallagher, et al., 2013 [52]	To assess family members’ satisfaction with end-of-life care, focusing on the last 48 h of life, and to examine how perceptions of care differed across various care settings.	Cross-sectional observational survey.	Participants were identified through a regional health authority database of deaths.	90 participants	After-Death Bereaved Family Member Interview
Burge, et al., 2014 [27]	To examine bereaved family members’ perceptions of patient-focused, family-centered end-of-life care during the last 30 days of life, and to determine whether these perceptions differed according to the location of care.	Population-based cross-sectional mortality follow-back survey.	Potential informants were identified using the ‘informant’ field on the death certificate of all adults.	1358 participants	After-Death Bereaved Family Member Interview
Hunt, et al., 2014 [33]	To examine the quality of end-of-life care in England, determine whether patients died in their preferred place, and identify factors associated with achieving the preferred place of death using bereaved relatives’ reports.	Population-based cross-sectional mortality follow-back survey.	A stratified sampling strategy was used to assign potential participants.	22,661 participants	Views of Informal Carers—Evaluation of Services Short Form
Mayland, et al., 2014 [34]	To adapt and validate the Care Of the Dying Evaluation (CODE) questionnaire for use with bereaved relatives of patients who died at home.	Cross-sectional questionnaire validation study (psychometric validation).	Participants were identified from the Preferred Place of Care database.	72 participants	Care Of the Dying Evaluation
Roza, et al., 2015 [35]	To assess bereaved family members’ perceptions of the quality of end-of-life care provided on an acute inpatient palliative care unit.	Cross-sectional observational survey.	Eligible respondents were listed as the primary contact in the electronic medical record.	275 participants	Bereaved Family Survey

## Appendix B

**Table A2:** Analysis of Instruments (unpublished data).

Instrument	Country of Origin	Structural Dimensions	Item Count	Recruitment Methodologies	Timing of Post-Death Administration
Toolkit of Instruments to Measure End-of-Life Care [31]	USA	7 domains	38 items	Context: All care settings Method of administration: Telephone Reference period: Last 30 days of life	9 to 15 months after death
Family Assessment of Treatment at End of Life Survey [32]	USA	9 domains	32 items	Context: Veterans Affairs System Method of administration: Telephone Reference period: Last 30 days of life	6 weeks after death
Client-Centered Care Questionnaire [53]	Canada (Ontario)	-	15 items	Context: Home care services by a Community Care Access Centre Method of administration: Postal questionnaire Reference period: Not available	Not available
Views of Informal Carers—Evaluation of Services Short Form [33]	England	8 domains	58 items	Context: All care settings Method of administration: Postal questionnaire Reference period: Last 3 Months of Life	6 to 12 months after death
Care Of the Dying Evaluation [34]	England	7 domains	40 items	Context: Home care Method of administration: Postal questionnaire Reference period: Last days of life	2 months after death
After-Death Bereaved Family Member Interview [27]	Canada (Nova Scotia)	6 domains	21 items	Context: Home, Hospital, Palliative care unit, Long-term care facility Method of administration: Postal questionnaire, toll-free telephone, email Reference period: Last 30 days of life	3 to 7 months prior to the start date of the wave
Bereaved Family Survey [35]	USA	-	12 items	Context: Palliative Care Method of administration: Postal questionnaire and telephone Reference period: Last admission	8 weeks after death
